# Supplementary material for: Filtration rates of the manila clam, Ruditapes philippinarum, in tidal flats with different hydrographic regimes
Source: PLoS One. 2020 Feb 10;15(2):e0228873. doi: 10.1371/journal.pone.0228873 (PMC7010307; doi:10.1371/journal.pone.0228873)
Supplement: S1 Table — (DOCX) [file pone.0228873.s001.docx]

Table S1. Variations on mean concentration of SS and POM in two experimental chambers and seawater over the entire study period at Geunso and Sihwa tidal flats.

|  | | 1st  (10:30 for Geunso and 13:30 for Sihwa) | | | | | 2nd  (12:00 for Geunso and 14:30 for Sihwa) | | | | | 3rd  (13:40 for Geunso and 15:30 for Sihwa) | | | | | 4th  (15:00 for Geunso and 16:30 for Sihwa) | | | | | 5th  (16:40 for Geunso) | | | |
| --- | --- | --- | --- | --- | --- | --- | --- | --- | --- | --- | --- | --- | --- | --- | --- | --- | --- | --- | --- | --- | --- | --- | --- | --- | --- |
|  |  | SS (mg L^-1^) | POC  (mg L^-1^) | PON  (mg L^-1^) | Chl *a*  (mg L^-1^) | SS (mg L^-1^) | | POC  (mg L^-1^) | PON  (mg L^-1^) | Chl *a*  (mg L^-1^) | SS (mg L^-1^) | | POC  (mg L^-1^) | PON  (mg L^-1^) | Chl *a*  (mg L^-1^) | SS (mg L^-1^) | | POC  (mg L^-1^) | PON  (mg L^-1^) | Chl *a*  (mg L^-1^) | SS (mg L^-1^) | | POC  (mg L^-1^) | PON  (mg L^-1^) | Chl *a*  (mg L^-1^) |
| Geunso | *Ruditapes* chamber | 0.13±0.00 | 5.38±1.05 | 0.34±0.12 | 5.55±1.21 | 0.06±0.01 | | 2.49±0.15 | 0.15±0.03 | 2.36±0.64 | 0.04±0.00 | | 1.79±0.10 | 0.10±0.01 | 1.37±0.46 | 0.02±0.01 | | 0.98±0.30 | 0.07±0.02 | 1.44±0.65 | 0.01±0.00 | | 0.90±0.13 | 0.06±0.01 | 1.60±0.64 |
|  | Control chamber | 0.14±0.03 | 5.25±0.73 | 0.33±0.03 | 6.17±1.96 | 0.04±0.00 | | 2.45±0.41 | 0.15±0.01 | 2.56±1.07 | 0.03±0.00 | | 2.42±0.07 | 0.14±0.01 | 2.64±0.49 | 0.02±0.00 | | 2.56±0.12 | 0.14±0.01 | 4.14±0.41 | 0.01±0.00 | | 2.23±0.33 | 0.13±0.01 | 5.30±0.21 |
|  | p value | 0.78 | 0.94 | 0.98 | 0.61 | 0.51 | | 0.90 | 0.68 | 0.67 | <0.05 | | 0.11 | 0.24 | <0.05 | 0.79 | | 0.13 | 0.14 | <0.05 | 0.37 | | 0.32 | <0.05 | <0.05 |
|  | Seawater | 0.07 | 6.56 | 0.44 | 4.79 | 0.03 | | 2.03 | 0.09 | 1.62 | 0.01 | | 1.00 | 0.05 | 2.25 | 0.02 | | 1.11 | 0.06 | 2.25 | 0.05 | | 2.54 | 0.19 | 6.10 |
| Sihwa | *Ruditapes* chamber | 0.10±0.00 | 2.41±0.28 | 0.22±0.02 | 3.35±0.86 | 0.09±0.01 | | 2.15±0.24 | 0.16±0.00 | 2.78±0.10 | 0.02±0.00 | | 1.28±0.20 | 0.09±0.03 | 1.33±0.57 | 0.01±0.00 | | 0.89±0.27 | 0.08±0.02 | 1.49±0.25 | - | | - | - | - |
|  | Control chamber | 0.09±0.03 | 2.51±0.37 | 0.23±0.01 | 3.27±0.85 | 0.08±0.02 | | 2.57±0.13 | 0.23±0.01 | 3.24±0.41 | 0.08±0.02 | | 2.43±0.00 | 0.22±0.02 | 3.25±0.44 | 0.08±0.02 | | 2.57±0.43 | 0.22±0.00 | 4.54±0.34 | - | | - | - | - |
|  | p value | 0.14 | <0.05 | 0.12 | <0.05 | <0.05 | | <0.05 | <0.05 | 0.18 | 0.29 | | <0.05 | <0.05 | <0.05 | 0.19 | | 0.15 | <0.05 | <0.05 |  | |  |  |  |
|  | Seawater | 0.08 | 2.18 | 0.14 | 2.37 | 0.10 | | 1.19 | 0.07 | 1.77 | 0.08 | | 0.87 | 0.05 | 2.16 | 0.07 | | 1.68 | 0.08 | 1.97 | - | | - | - | - |

Significant differences by *t* test at 0.05
