# Supplementary material for: Filtration rates of the manila clam, Ruditapes philippinarum, in tidal flats with different hydrographic regimes
Source: PLoS One. 2020 Feb 10;15(2):e0228873. doi: 10.1371/journal.pone.0228873 (PMC7010307; doi:10.1371/journal.pone.0228873)
Supplement: S2 Table — (DOCX) [file pone.0228873.s002.docx]

Table S2. Comparison of filtration rate of *Ruditapes philippinarum* for POM between Geunso and Sihwa tidal flats over time.

|  | Filtration rate (L h^-1^ gDW^-1^) | | | | | | | | | | | |
| --- | --- | --- | --- | --- | --- | --- | --- | --- | --- | --- | --- | --- |
|  | 1st  (10:30-12:00 for Geunso and 13:30-14:30 for Sihwa) | | | 2nd  (12:00-13:40 for Geunso and 14:30-15:30 for Sihwa) | | | 3rd  (13:40-15:00 for Geunso and 15:30-16:30 for Sihwa) | | | 4th  (15:00-16:40 for Geunso) | | |
|  | POC | PON | Chl *a* | POC | PON | Chl *a* | POC | PON | Chl *a* | POC | PON | Chl *a* |
| Geunso | -0.19 ±1.81 | -0.53 ±2.47 | -0.30 ±0.76 | 0.38±0.58 | 0.17±0.13 | 2.54±1.61 | 0.61±0.04 | 0.89±0.19 | 0.25±0.35 | -0.88 ±1.33 | -0.24 ±0.71 | 3.17±1.72 |
| Sihwa | 0.43±1.09 | 0.43±2.25 | -10.79 ±6.76 | 1.36±2.55 | 2.29±1.85 | 5.46±1.07 | 2.86±1.28 | 0.51±1.02 | -2.04 ±2.88 | - | - | - |
