# Supplementary material for: Filtration rates of the manila clam, Ruditapes philippinarum, in tidal flats with different hydrographic regimes
Source: PLoS One. 2020 Feb 10;15(2):e0228873. doi: 10.1371/journal.pone.0228873 (PMC7010307; doi:10.1371/journal.pone.0228873)
Supplement: Attachment — Submitted filename: Response to reviewers comments.docx [file pone.0228873.s004.docx]

Reviewer #1: As I wrote before, it is not appropriate to combine measurements of three different components of the seston (POC, PON, chla), all collected in the same chamber at the same time, to calculate a mean filtration rate. Mean filtration rate could be based on the filtration of the same component of the water across the two experimental chambers at each site, after each is adjusted for the change in the companion reference chamber. A t-test could then be done using two samples at each site, since there were two experimental chambers (containing clams) at each site. (Note: I am actually uncertain whether the “four chambers” on line 155 refer to four chambers per tidal flat, or four chambers in total. If the latter, then there is not a possibility to compare the two tidal flats statistically, as there is only one filtration value for each flat. But in their response the authors write “two experimental chambers represent replicates” so I think they should be able to do statistics with n=2 at each tidal flat.)

→ The mean filtration rate (three different components of seston) was deleted according to your suggestion. The four chambers represent two experimental chambers (with clams) and two control chamber (without clams) at each site. Thus, we have done t-test using two samples at each site.

Line 230: How were the control chambers used in the calculations of filtration rate? The authors write: “the filtration rate was calculated for the period in which the maximum slope of the decreasing indicator concentration was observed.” There are three issues to address: 1) The maximum slope at Sihwa (Fig 5) occurs in the first time interval, but is equivalent for clam and reference chambers, such that no net filtration would be indicated IF the values were properly adjusted by comparison with reference; 2) the maximum slope seems to indicate the steepest portion of Figures 5 and 6, rather than the portion with the largest exponent in the equation for filtration; 3) selecting a time of maximum filtration from among the time periods creates potential bias if clam filtration at one site is more variable than at the other. The authors should do the following: 1) determine which reference chamber to pair with each clam chamber, 2) adjust Ct in the clam chamber so that it accounts for change in the reference chamber (add C0-Ct in the reference chamber to Ct in the clam chamber), 3) determine Z for each time interval, 4) select maximum Z to calculate FR (since the authors indicate that they think maximum filtration is the best metric of filtration rate), 5) calculate Z for the entire 4 hours as another way to compare between the two tidal flats. After this process has been done, then the authors can carry out t-tests on n=2 samples per site, for each of the components in the water, and for maximum and 4-hour filtration rates.

→ The filtration was recalculated according to your suggestion (Table 3). The experimental chambers (with clams) were paired up with each control chamber (C1-R1/C2-R2, Fig. 2) and then Ct value of experimental chambers was adjusted by each control chamber value (C0-Ct). The maximum Z among time periods was selected and then filtration rate was calculated. A t-test was carried out based on two samples at each site as well as each component (POC, PON and Chl *a*). The filtration rate during entire experimental period was described in Table S2.

Table 2: Fresh weight is always a wet weight. So Fresh dry weight does not make sense. Are these values the Meat dry weights? Also on line 189.

→ It was corrected to flesh dry weight in Table 2 and manuscript.

Error bars on the figures: Thank you for specifying that the error bars represent 95% CI, but that does not address the whole issue I brought up last review. Is this the 95% CI based on n=2 (number of chambers), and if not, what is considered a sample?

→ The statistical analysis was conducted based on two samples as we noted above.

Line 29: Rewrite – Clams were smaller at Sihwa than Geunso sites, but the filtration rate of 50 clams per chamber was higher at Sihwa than Geunso. This difference was probably not due to the immediate environmental conditions, since the enclosed chambers experienced no net current or tidal exchange during the 4-hour monitoring interval, and the initial seston and chlorophyll concentrations in the chambers were similar. Instead, these filtration rates may differ due to the hydrographic regime, since Sihwa tides are limited by sluice gates. Generally, at Geunso relative to Sihwa, current speeds are faster, and submergence times are shorter. Sihwa typically has higher chlorophyll a concentrations, as well as better food quality based on C/N ratio of POM (Sihwa 12.8, Geunso 15.6). The endogenous circatidal rhythm…

I am not necessarily convinced that the sentence about endogenous circatidal rhythm follows from the data, since there are no data showing that filtration rate changes over time more at Geunso than at Sihwa.

→ It was corrected as follows : The filtration rate of clams for POM at Sihwa tidal flat (2.86 for POC, 2.29 for PON and 5.46 L h-1 gDW-1 for Chl a) was higher than that at Geunso tidal flat (0.61 for POC, 0.89 for PON and 2.54 L h-1 gDW-1 for Chl *a*) which resulted from differences in the hydrographic regime, including tide characteristics, current speed and submergence time, and food quantity and quality (L-29). The description of endogenous circatidal rhythm was deleted in the manuscript.

Line 39: There is no information about the effects of hydrographic regime, which implies that causality has been tested. This sentence should be rewritten: These findings suggest that hydrographic regime could be important in understanding in situ filtration rates.

→ It was corrected according to your suggestion as follows : These findings suggest that hydrographic regime could be important in understanding *in situ* filtration rates of *R. philippinarum*.
