# Supplementary material for: Filtration rates of the manila clam, Ruditapes philippinarum, in tidal flats with different hydrographic regimes
Source: PLoS One. 2020 Feb 10;15(2):e0228873. doi: 10.1371/journal.pone.0228873 (PMC7010307; doi:10.1371/journal.pone.0228873)
Supplement: Attachment — Submitted filename: Response to reviewers comments.docx [file pone.0228873.s006.docx]

→ It was corrected as follows : Dissolved oxygen was monitored to prevent a decrease in metabolic activity due to depletion of dissolved oxygen in the chamber, and when the concentration decreased below 80%, oxygen was supplied using an aerator installed inside the chamber (L141-143).

→ 500 ml is correct volume. We did not use all water samples (750 ml except 250 ml for chlorophyll *a* analysis) for SS, POC and PON analysis (L 205).

→ It was corrected as follows : Thereafter, no significant changes in the chlorophyll *a* concentration in the Ruditapes chamber occurred, while that in the control chamber tended to increase (Table S1, L359-361).

→ It was corrected as follows : Han et al. (2008) reported that the filtration rate of small *Ruditapes philippinarum* (0.2 gDW) was about 28% higher than that of large ones (0.4 gDW) at 20 °C. This indicated that the filtration rate of *Ruditapes* can be changed by size and biomass, but, the difference in filtration rate is too great to be explained by size and biomass in this study (L 404-407)

→ It was corrected as follows : One experimental condition that must be considered carefully when calculating the amount of POM removed from seawater by filter feeders using a closed chamber, as indicated in this study, is that the concentration of POM introduced into the chamber at the beginning of the experiment should be maintained (L408-411).

→ It was corrected as follows : The relatively high filtration rate at Sihwa tidal flat can be attributed to this difference in hydrographic regimes. Another factor affecting the difference in filtration rate is high productivity in the water column at Sihwa tidal flat, which causes a quantitative difference in the food available to Manila clams. The Manila clams at Sihwa tidal flat had a higher growth rate than those at Geunso tidal flat, and the mean chlorophyll a concentration in the water column was significantly higher at the former site than the latter, with values of 3.1 and 52.5 μg L^-1^, respectively (L435-440) .

→ It was corrected as follows : The filtration rates obtained in this study, especially at Sihwa tidal flat, were higher than those reported in previous studies. While Lee [21] in similar field experimental settings as the current study reported the filtration rate of the Manila clams ranging from 0.13 to 0.97 L h^-1^ gDW^-1^, our study reports significantly higher values (2.29 to 5.46 L h^-1^ gDW^-1^), which could be related to differences in experiment methods. This value is significantly lower than the experiment results of the present study (L452-456).

→ It was corrected as follows : In another field experiment, Aoyama and Suzuki [23] reported the filtration rate of 3.44 L h^-1^ gDW^-1^ (L 464-465).

→ It was corrected as follows : In conclusion, this study evaluated differences in the filtration rate of *R. philippinarum* due to differing hydrographic regimes between two tidal flats that the influence of differing hydrographic conditions on the filtration rates of Manila clams has not been studied previously. Though this study was limited by factors such as salinity, turbidity and particle size that affect variations in filtration rates, the findings provide important information about the effects of hydrographic regime on the filtration rate of this species (L472-475).

→ We added this sentence : We would like to thank Dr. Jennifer Ruesink and anonymous reviewer for their valuable comments.
